# Supplementary material for: Interaction proteome of human Hippo signaling: modular control of the co‐activator YAP1
Source: Mol Syst Biol. 2013 Dec 20;9:713. doi: 10.1002/msb.201304750 (PMC4019981; doi:10.1002/msb.201304750)
Supplement: Supplementary file 1 — Supplementary Figure 1 [file MSB-9-1-713-s01.pdf]

Supplementary Results

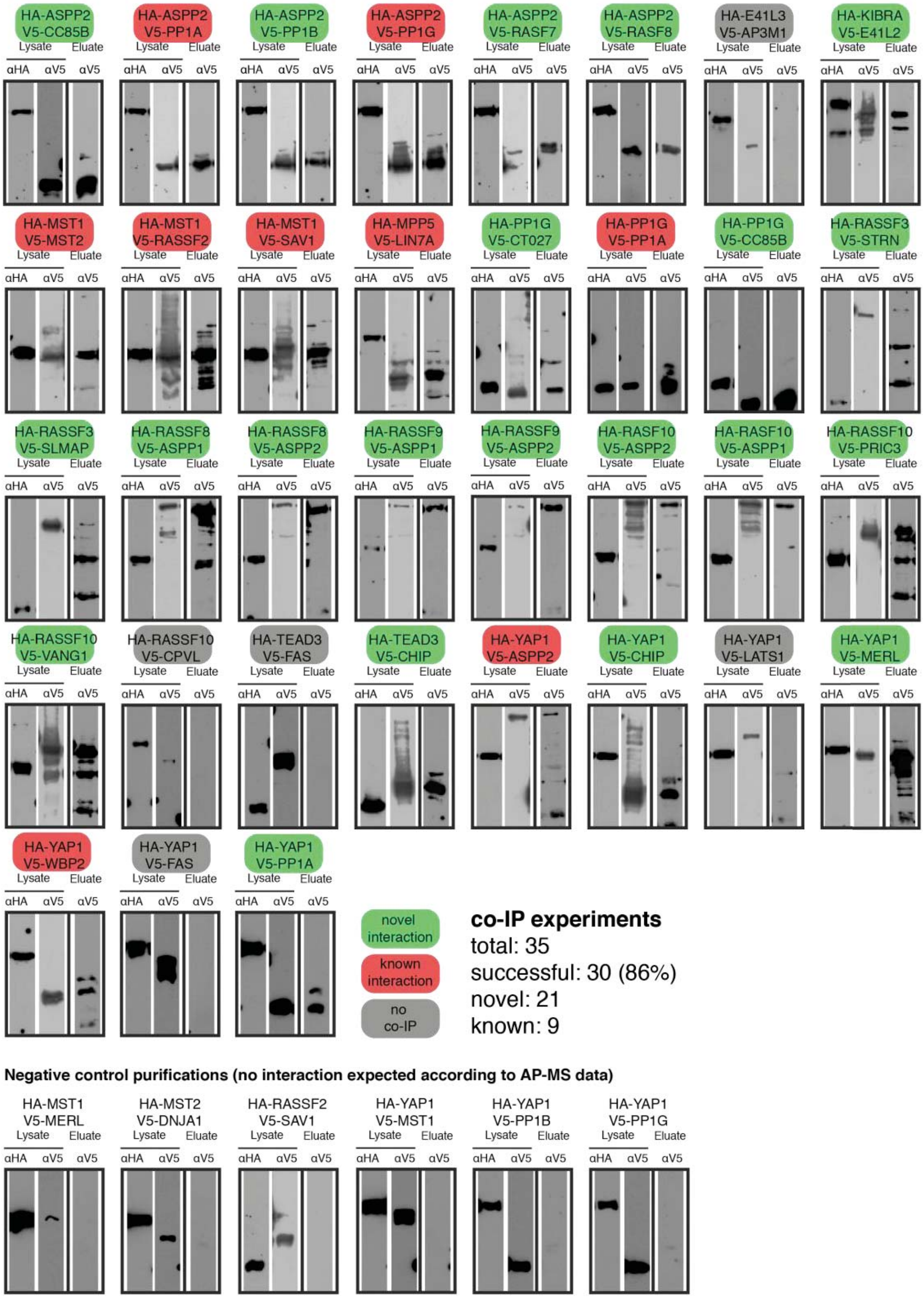

Supplementary Figure S1: Experimental validation of AP-MS results by co-immunprecipitation Western blot analysis.

Co-immunoprecipitation experiments of interacting proteins detected by AP-MS in this study were co-expressed as HA and V5 tagged versions in HEK-293 cells and purified by anti-HA immunoprecipitation. Expression of the HA and the V5 constructs was tested from the lysates with anti-HA and anti-V5 antibodies. After HA purification, the eluates were blotted and probed with anti-V5 antibody. Out of 35 experiments, 21 novel (green) and 9 known (red) interactions could be validated. As negative control, random pairs of network components were co-expressed and did not co-purify (bottom row).
