# Supplementary material for: Interaction proteome of human Hippo signaling: modular control of the co‐activator YAP1
Source: Mol Syst Biol. 2013 Dec 20;9:713. doi: 10.1002/msb.201304750 (PMC4019981; doi:10.1002/msb.201304750)
Supplement: Supplementary file 2 — Supplementary Figure 2 [file MSB-9-1-713-s02.pdf]

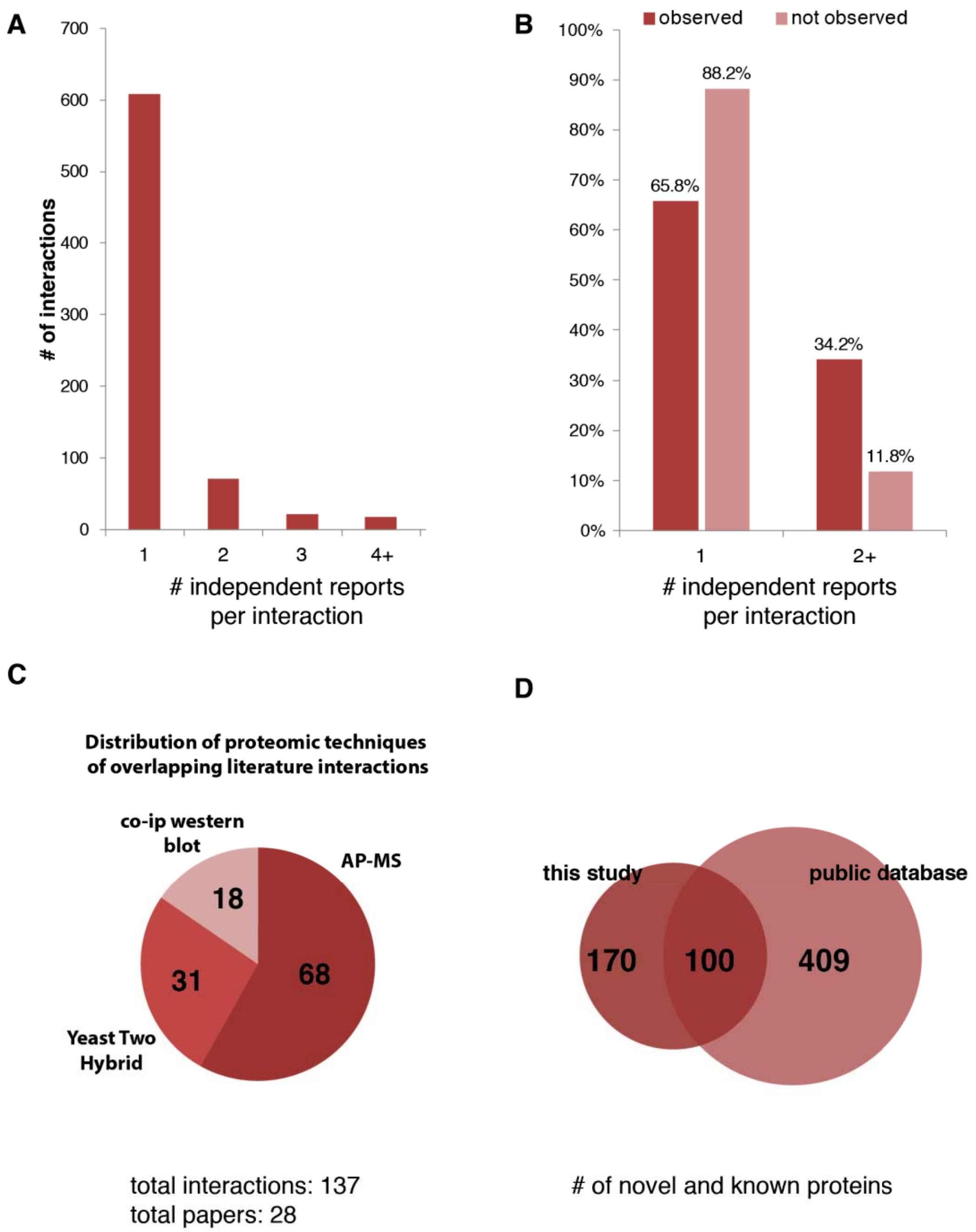

Supplementary Figure S2: Comparison of AP-MS data to publicly available protein interaction data.

(A) Distribution of number of independent reports (by PubMed identifier) across all extracted interactions. Publicly annotated protein interactions were extracted from the protein interaction network analysis platform (PINA) for the baits used in this study. A large majority (84.6%) of public protein interactions is reported by a single publication. (B) The fraction of robust public interactions supported by more than one publication is higher among the interactions observed also in the presented AP-MS study as compared to the

interactions not observed. All interactions from this study that match to the PINA dataset were compared to PINA exclusive interactions with respect to the number of independent publications supporting an interaction. There was a threefold enrichment in interactions observed multiple times in the literature in the presented AP-MS compared to the group of interactions not observed in our study. (C) Distribution of detection methods for public interactions. A total of 28 different publications are needed to cover the overlap of 137 interactions with this study. (D) Comparison of experimentally identified HCIPs and already know interactors for the baits used in this study.
