# Supplementary material for: Interaction proteome of human Hippo signaling: modular control of the co‐activator YAP1
Source: Mol Syst Biol. 2013 Dec 20;9:713. doi: 10.1002/msb.201304750 (PMC4019981; doi:10.1002/msb.201304750)
Supplement: Supplementary file 3 — Supplementary Figure 3 [file MSB-9-1-713-s03.pdf]

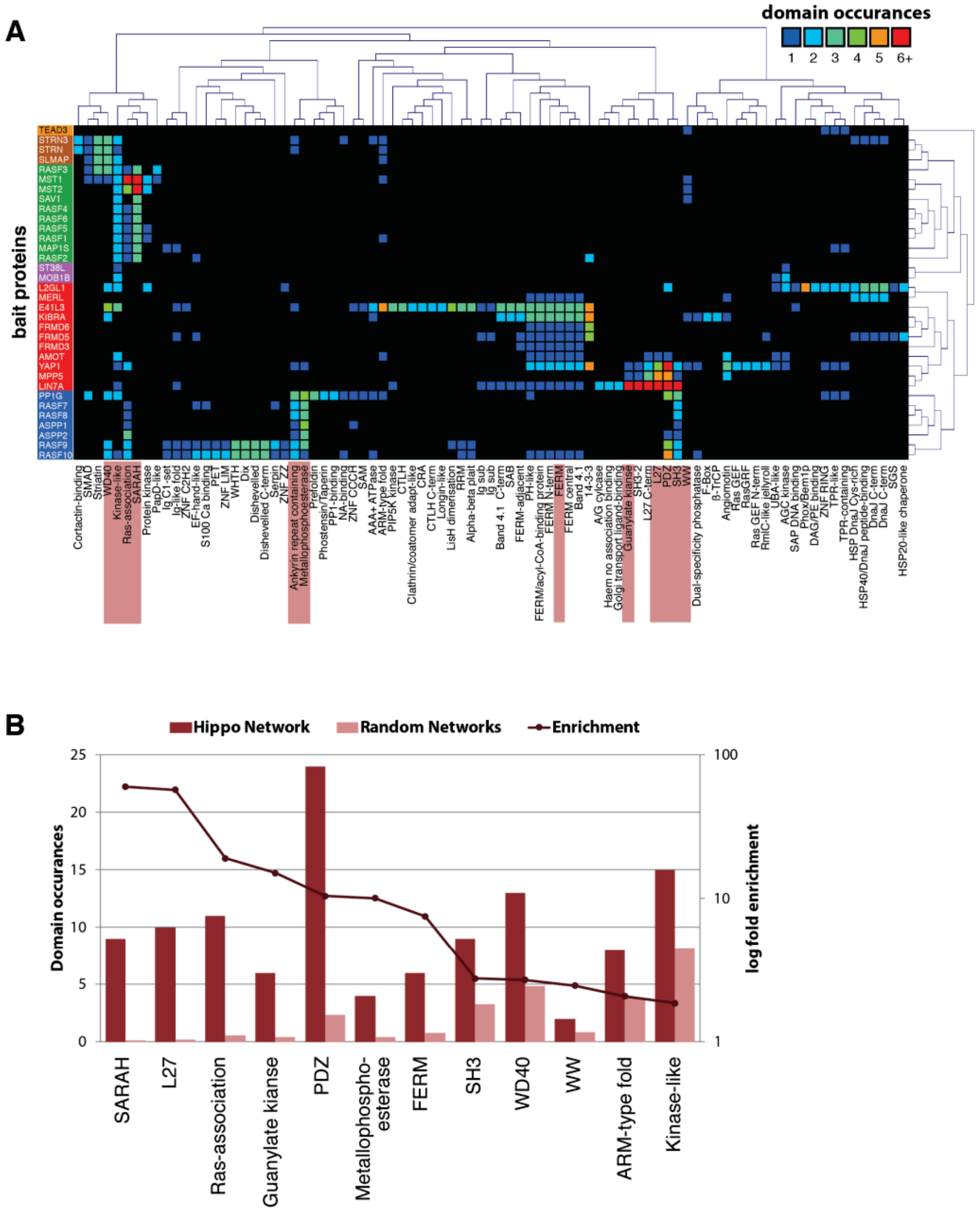

Supplementary Figure S3: Domain distributions and enrichment of identified Hpo pathway proteins.

(A) Bait proteins and domains identified in the respective bait purification were hierarchically clustered by an uncentered Pearson correlation algorithm. (B) Enrichment of a subset of prominent domains compared to random protein networks of the same size. 270 proteins were sampled randomly 10,000 times from the canonical human Swiss-Prot database (complete proteome dataset) and used to calculate the domain enrichment in the presented AP-MS data.
