# Supplementary material for: Interaction proteome of human Hippo signaling: modular control of the co‐activator YAP1
Source: Mol Syst Biol. 2013 Dec 20;9:713. doi: 10.1002/msb.201304750 (PMC4019981; doi:10.1002/msb.201304750)
Supplement: Supplementary file 4 — Supplementary Figure 4 [file MSB-9-1-713-s04.pdf]

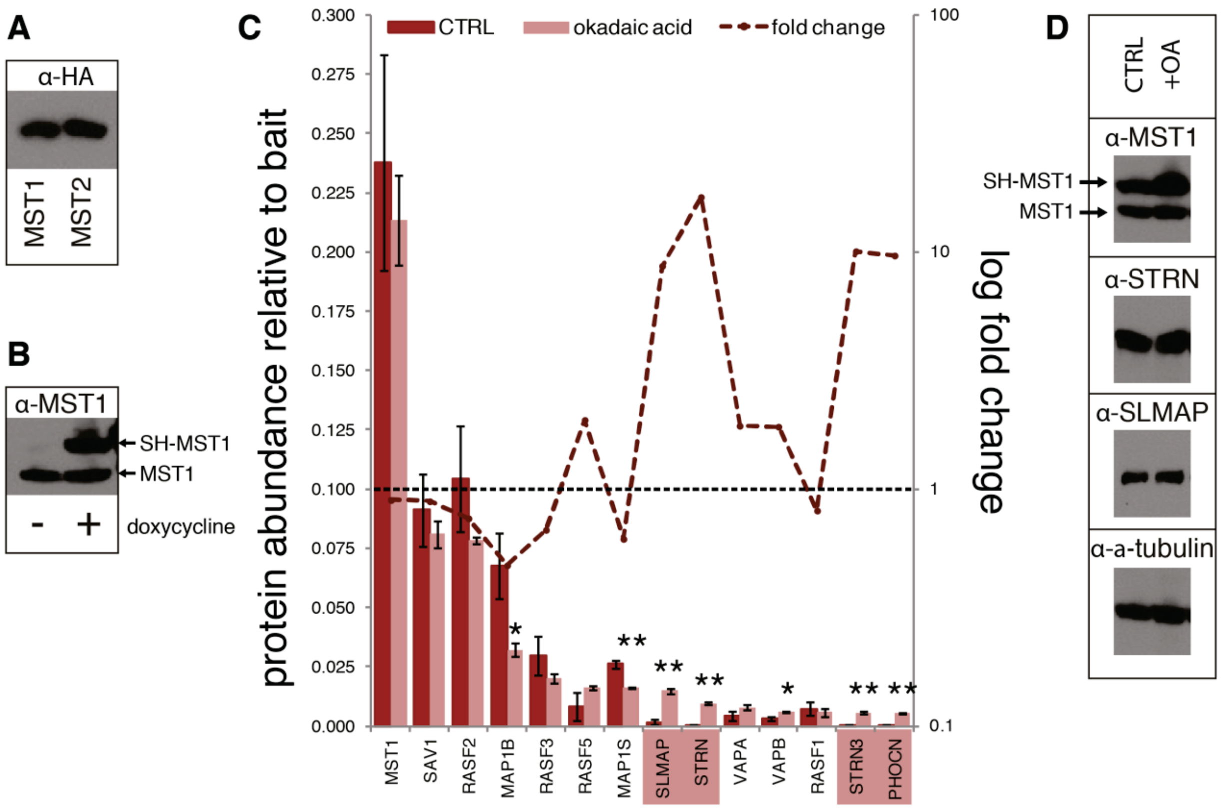

Supplementary Figure S4: MST2 binds to STRIPAK components following okadaic acid treatment.

(A) Western blot analysis of exogenous SH-MST1 and SH-MST2 with anti-HA antibody. (B) Western blot analysis of endogenous MST1 and exogenous SH-MST1 protein with anti-MST1 antibody. (C) Abundance changes of interacting proteins of MST2 upon okadaic acid stimulation. HEK293 cells expressing Strep-HA tagged MST2 were treated with 100 nM okadaic acid (OA) for 2h. Left axis represents the protein abundance relative to MST2. Right axis (log fold change; dotted line) is the logarithmic fold change of the relative abundance of proteins bound to MST2, following OA treatment. Similar to MST1 (Figure 3C), MST2 complexes contained an increased amount of STRIPAK (protein names highlighted in red) associated proteins, whereas SARAH module components only showed marginal changes. Error bars indicate standard deviation from biological triplicates. Asterisks indicate t-test statistical significance (* $p < 0.05$; ** $p < 0.01$). (D) Effects of okadaic acid treatment on expression levels of MST1, STRN and SLMAP (probed with corresponding antibody for the endogenous proteins).
