# Supplementary material for: Interaction proteome of human Hippo signaling: modular control of the co‐activator YAP1
Source: Mol Syst Biol. 2013 Dec 20;9:713. doi: 10.1002/msb.201304750 (PMC4019981; doi:10.1002/msb.201304750)
Supplement: Supplementary file 5 — Supplementary Figure 5 [file MSB-9-1-713-s05.pdf]

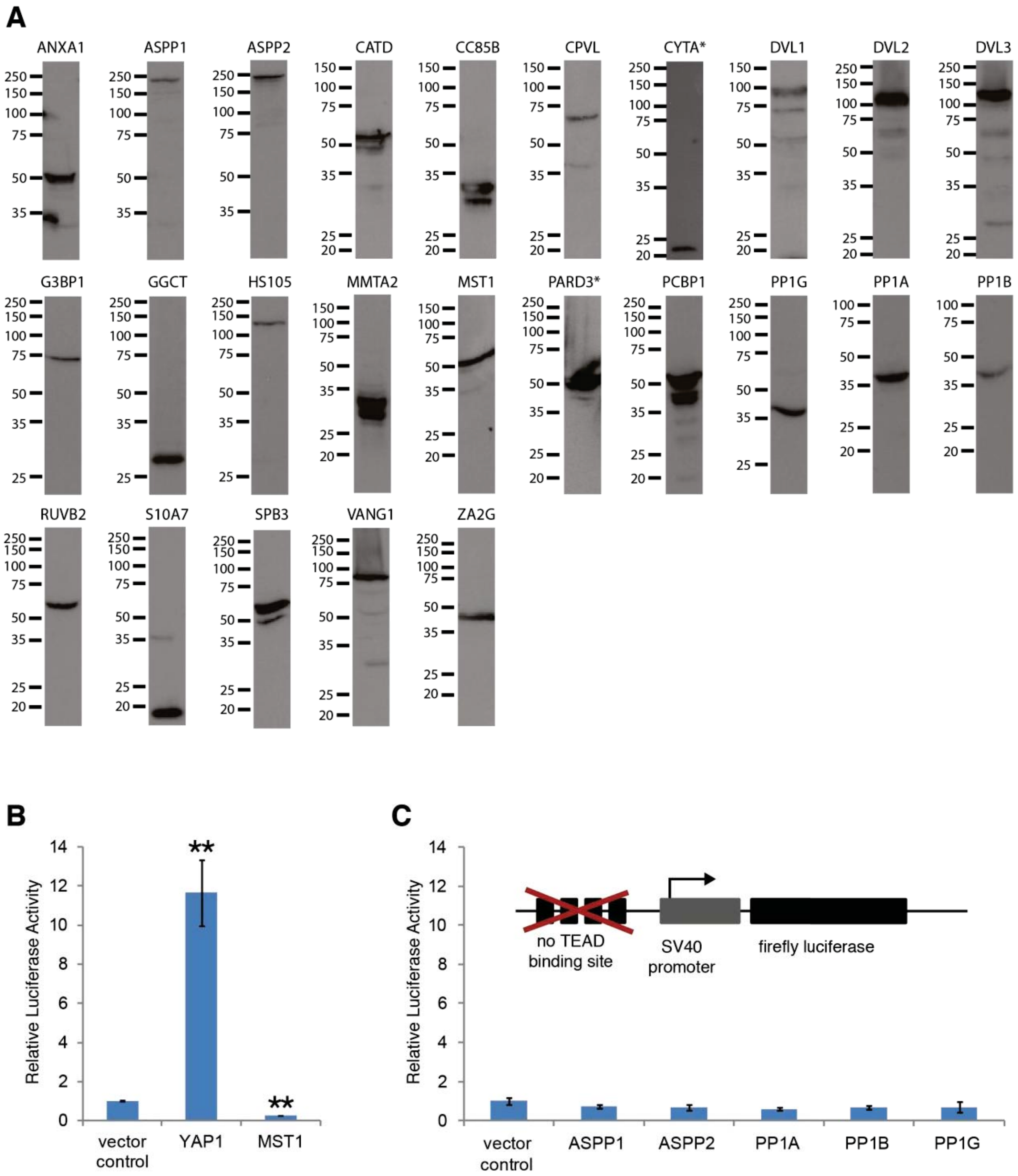

Supplementary Figure S5: Dual luciferase reporter assay of PP1-ASPP network components.

(A) Western blots of indicated Hpo network components expressed as V5-tagged proteins in transiently transfected HEK293-SH-YAP1 cells used for dual luciferase assays. Overexpressed proteins were detected with anti-V5 and anti-mouse-HRP antibodies. Asterisk (*) indicates longer exposure time. (B) Co-transfection of V5-YAP1 and V5-MST1 were used as positive and negative controls for the TEAD luciferase assay. Error bars

indicate standard deviation from biological triplicates. Asterisks indicate t-test statistical significance (* $p < 0.05$; ** $p < 0.01$). (C) ASPP2 and PP1A/G mediated transcriptional activation shown in Figure 4B require TEAD binding sites. Dual luciferase assay following transfection of indicated expression plasmids and pGL3-49 lacking TEAD binding sites.
