# Supplementary material for: Interaction proteome of human Hippo signaling: modular control of the co‐activator YAP1
Source: Mol Syst Biol. 2013 Dec 20;9:713. doi: 10.1002/msb.201304750 (PMC4019981; doi:10.1002/msb.201304750)
Supplement: Supplementary file 6 — Supplementary Figure 6 [file MSB-9-1-713-s06.pdf]

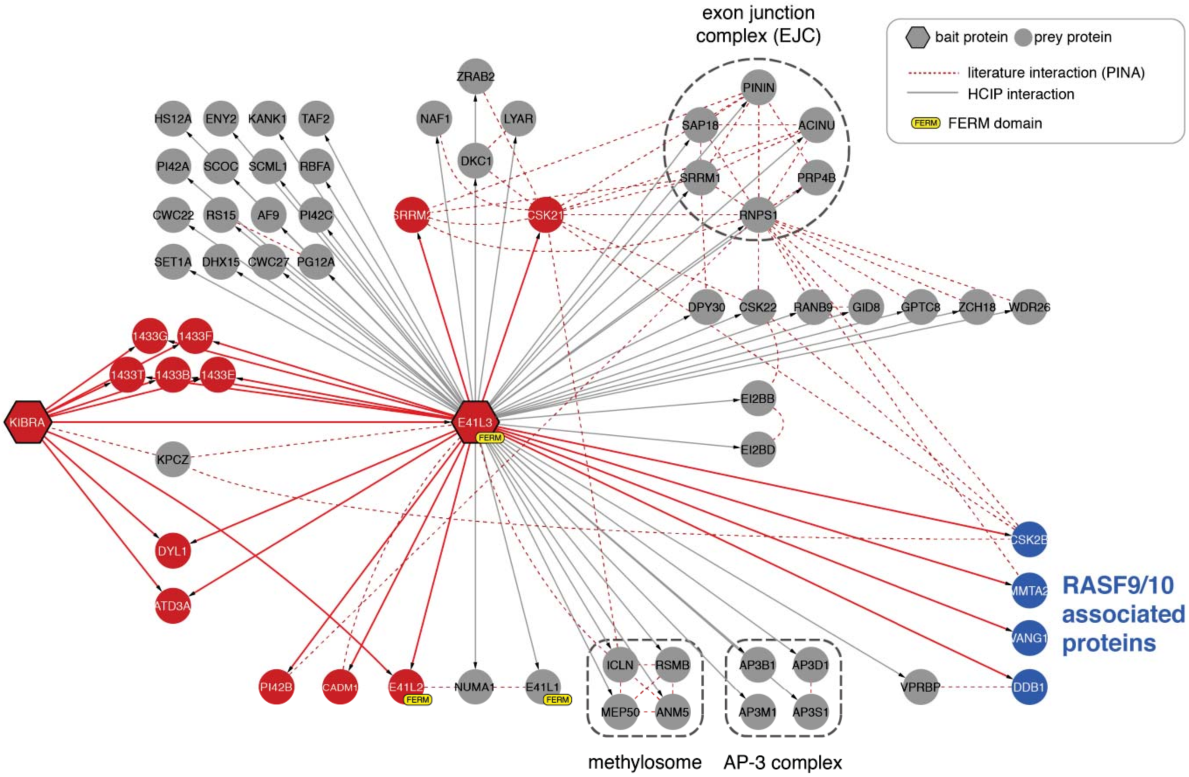

Supplementary Figure S6: The E41L3 interaction proteome.

E41L3 AP-MS revealed the most connected interactome of this study (62 HCIPs). E41L3 interacts with Hpo network component KIBRA and proteins shared with RASF9 and RASF10. Public protein interaction data for the identified prey proteins revealed associations of E41L3 with several protein complexes, such as the exon junction complex, the methylosome and the AP-3 complex. Node color corresponds to the partitioning in the modules defined in Figure 2. Proteins not allocated to specific modules are shown in grey.
