# Supplementary material for: Interaction proteome of human Hippo signaling: modular control of the co‐activator YAP1
Source: Mol Syst Biol. 2013 Dec 20;9:713. doi: 10.1002/msb.201304750 (PMC4019981; doi:10.1002/msb.201304750)
Supplement: Supplementary file 10 — Supplementary Materials‐Methods [file MSB-9-1-713-s10.pdf]

Supplementary Materials and Methods

Co-immunoprecipitation and western blotting analysis

For co-immunoprecipitation and Western blotting analysis, 5 x 10^5 HEK-293 ATCC® (LGC Standards GmbH) were co-transfected with HA-tagged and V5-tagged interacting proteins. After 24h of transient expression, the cells were lysed with 500 µl of lysis buffer (50 mM HEPES pH 7.5, 150 mM NaCl, 50 mM NaF, 0.5% Igepal CA-630 (NP-40 Substitute), 200 µM Na_3VO_4, 1 mM PMSF, and 1x Protease Inhibitor mix (Sigma)) and incubated on ice for 10 min. Insoluble material was removed by centrifugation. Cleared lysates were incubated with 30 µl HA11 agarose (Sigma) for 1h at 4°C on a tube rotator. The beads were washed three times with lysis buffer and eluted with SDS sample buffer. The samples were boiled at 95°C for 5 min and separated by gel electrophoresis on 10% SDS-PAGE gels. Western blot transfer was done on nitrocellulose membranes (Whatman GmbH) using a semi-dry western blotting system (Bio-Rad). Immunoprecipitated proteins were detected with the primary monoclonal anti-HA antibodies (HA.11, Covance Inc) and anti-V5 (Life Technologies), respectively. As secondary antibody horseradish peroxidase (HRP) conjugated anti-mouse HRP (Santa Cruz Biotechnology Inc) was used for detection by enhanced chemiluminescence (ECL; Bio-Rad).

Western blotting analysis of endogenous proteins

5 x 10^5 HEK-293 ATCC® (LGC Standards GmbH) were either left untreated or induced with 1.3 µg/ml doxycline for 24 h. Where indicated, cells were treated with 100 nM okadaic acid for 2 h. The cells were lysed with 500 µl of lysis buffer (50 mM HEPES pH 7.5, 150 mM NaCl, 50 mM NaF, 0.5% Igepal CA-630 (NP-40 Substitute), 200 µM Na_3VO_4, 1 mM PMSF, and 1x Protease Inhibitor mix (Sigma)) and incubated on ice for 10 min. Insoluble material was removed by centrifugation. The proteins from the cleared lysate were denatured with SDS sample buffer and by boiling at 95°C for 5 min, and separated by gel electrophoresis. Western blotting was done using a semi-dry blotting system (Bio-Rad). Endogenous proteins levels were detected with antibodies for MST1 (Cell Signaling; Cat#: 3682S), STRN (Santa Cruz Biotechnology Inc; Cat#: sc-136084), SLMAP (Santa Cruz Biotechnology Inc; Cat#: sc-100957) and α-Tubulin (Sigma Aldrich). As secondary antibody horseradish peroxidase (HRP) conjugated anti-mouse-HRP or anti-rabbit-HRP (Santa Cruz Biotechnology Inc) was used for detection by enhanced chemiluminescence (ECL; Bio-Rad).
