# Supplementary material for: Interaction proteome of human Hippo signaling: modular control of the co‐activator YAP1
Source: Mol Syst Biol. 2013 Dec 20;9:713. doi: 10.1002/msb.201304750 (PMC4019981; doi:10.1002/msb.201304750)
Supplement: Supplementary file 11 — Supplementary Table of Contents [file MSB-9-1-713-s11.pdf]

Supplementary information

Table of Content:

Supplementary Results **2**

Supplementary Figure S1: Experimental validation of AP-MS results by co-immunprecipitation Western blot analysis. 3

Supplementary Figure S2: Comparison of AP-MS data to publicly available protein interaction data. 4

Supplementary Figure S3: Domain distributions and enrichment of identified Hpo pathway proteins. 6

Supplementary Figure S4: MST2 binds to STRIPAK components following okadaic acid treatment. 7

Supplementary Figure S5: Dual luciferase reporter assay of PP1-ASPP network components 8

Supplementary Figure S6: The E41L3 interaction proteome. 10

Supplementary Materials and Methods **11**

Co-immunoprecipitation and western blotting analysis 11

Western blotting analysis of endogenous proteins 11

Supplementary Results

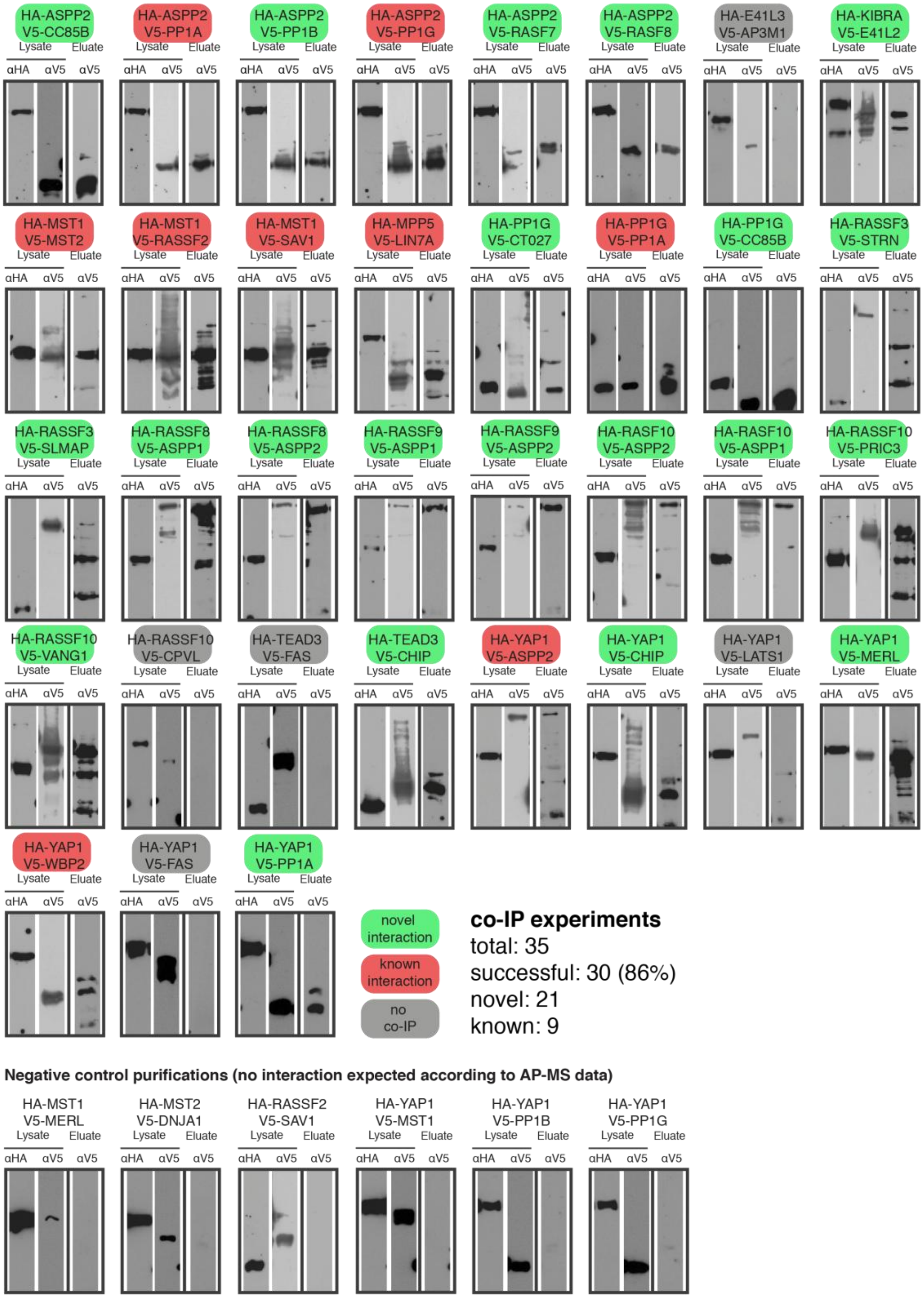

Supplementary Figure S1: Experimental validation of AP-MS results by co-immunprecipitation Western blot analysis.

Co-immunoprecipitation experiments of interacting proteins detected by AP-MS in this study were co-expressed as HA and V5 tagged versions in HEK-293 cells and purified by anti-HA immunoprecipitation. Expression of the HA and the V5 constructs was tested from the lysates with anti-HA and anti-V5 antibodies. After HA purification, the eluates were blotted and probed with anti-V5 antibody. Out of 35 experiments, 21 novel (green) and 9 known (red) interactions could be validated. As negative control, random pairs of network components were co-expressed and did not co-purify (bottom row).

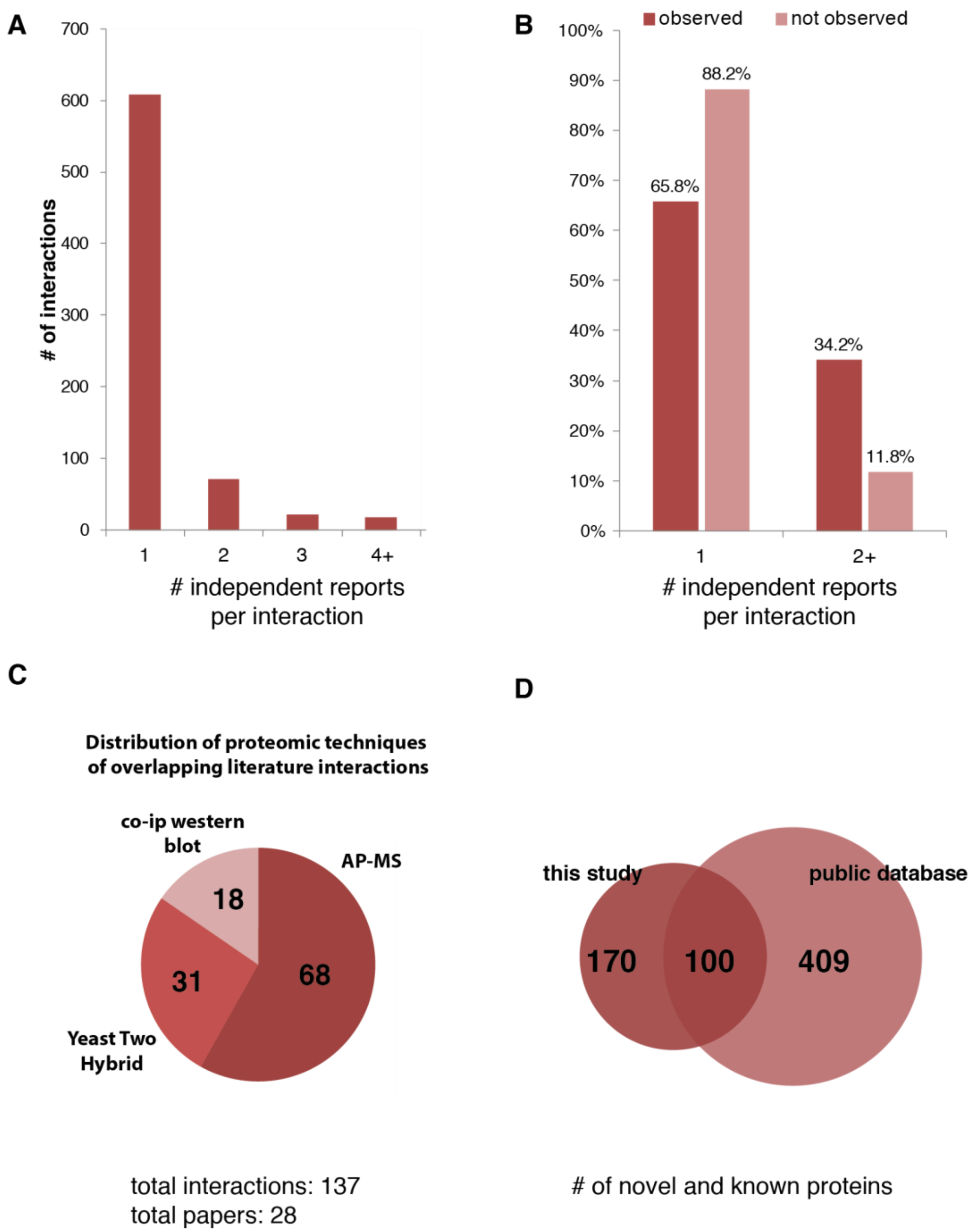

Supplementary Figure S2: Comparison of AP-MS data to publicly available protein interaction data.

(A) Distribution of number of independent reports (by PubMed identifier) across all extracted interactions. Publicly annotated protein interactions were extracted from the protein interaction network analysis platform (PINA) for the baits used in this study. A large majority (84.6%) of public protein interactions is reported by a single publication. (B) The fraction of robust public interactions supported by more than one publication is higher among the interactions observed also in the presented AP-MS study as compared to the

interactions not observed. All interactions from this study that match to the PINA dataset were compared to PINA exclusive interactions with respect to the number of independent publications supporting an interaction. There was a threefold enrichment in interactions observed multiple times in the literature in the presented AP-MS compared to the group of interactions not observed in our study. (C) Distribution of detection methods for public interactions. A total of 28 different publications are needed to cover the overlap of 137 interactions with this study. (D) Comparison of experimentally identified HCIPs and already know interactors for the baits used in this study.

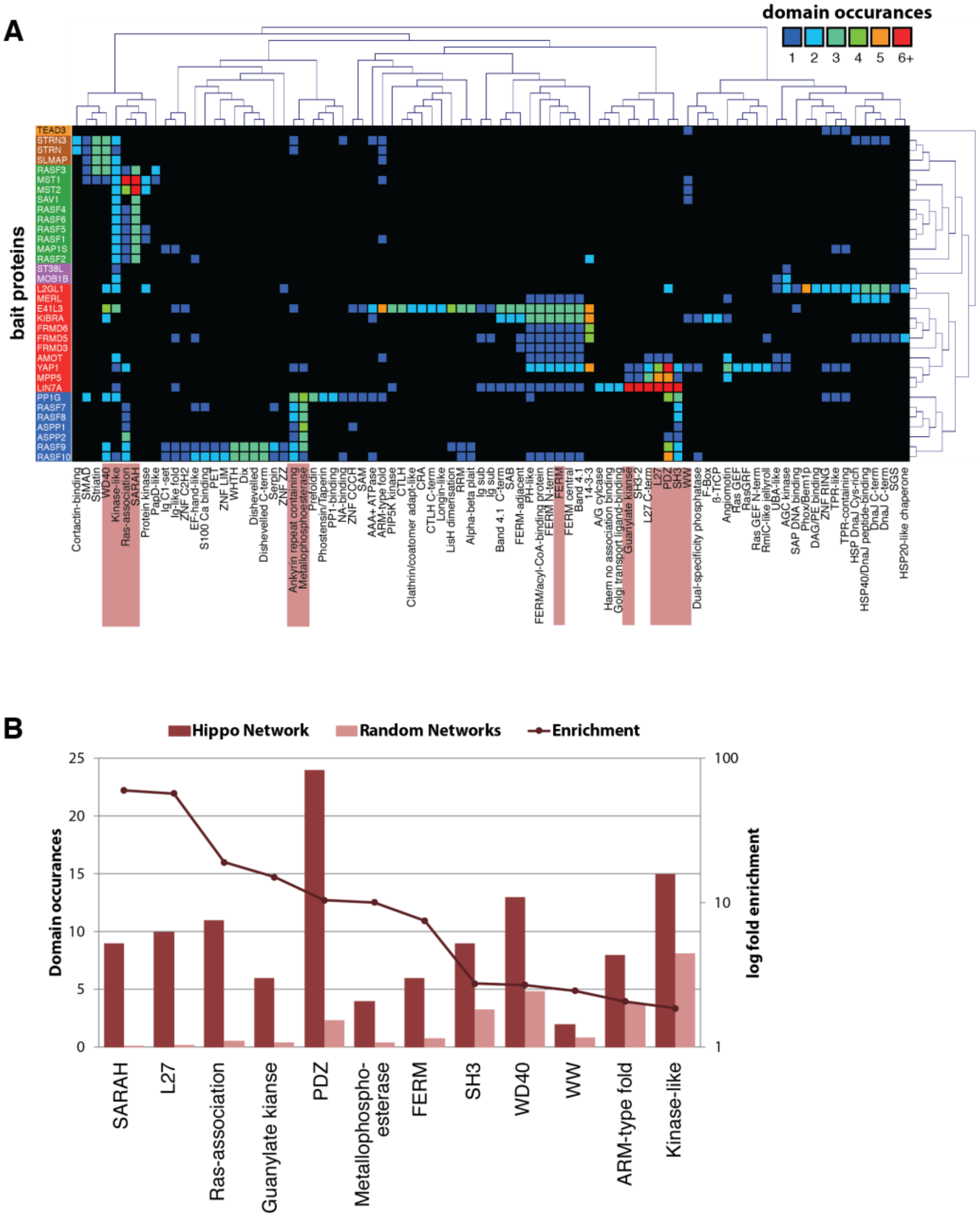

Supplementary Figure S3: Domain distributions and enrichment of identified Hpo pathway proteins.

(A) Bait proteins and domains identified in the respective bait purification were hierarchically clustered by an uncentered Pearson correlation algorithm. (B) Enrichment of a subset of prominent domains compared to random protein networks of the same size. 270 proteins were sampled randomly 10,000 times from the canonical human Swiss-Prot database (complete proteome dataset) and used to calculate the domain enrichment in the presented AP-MS data.

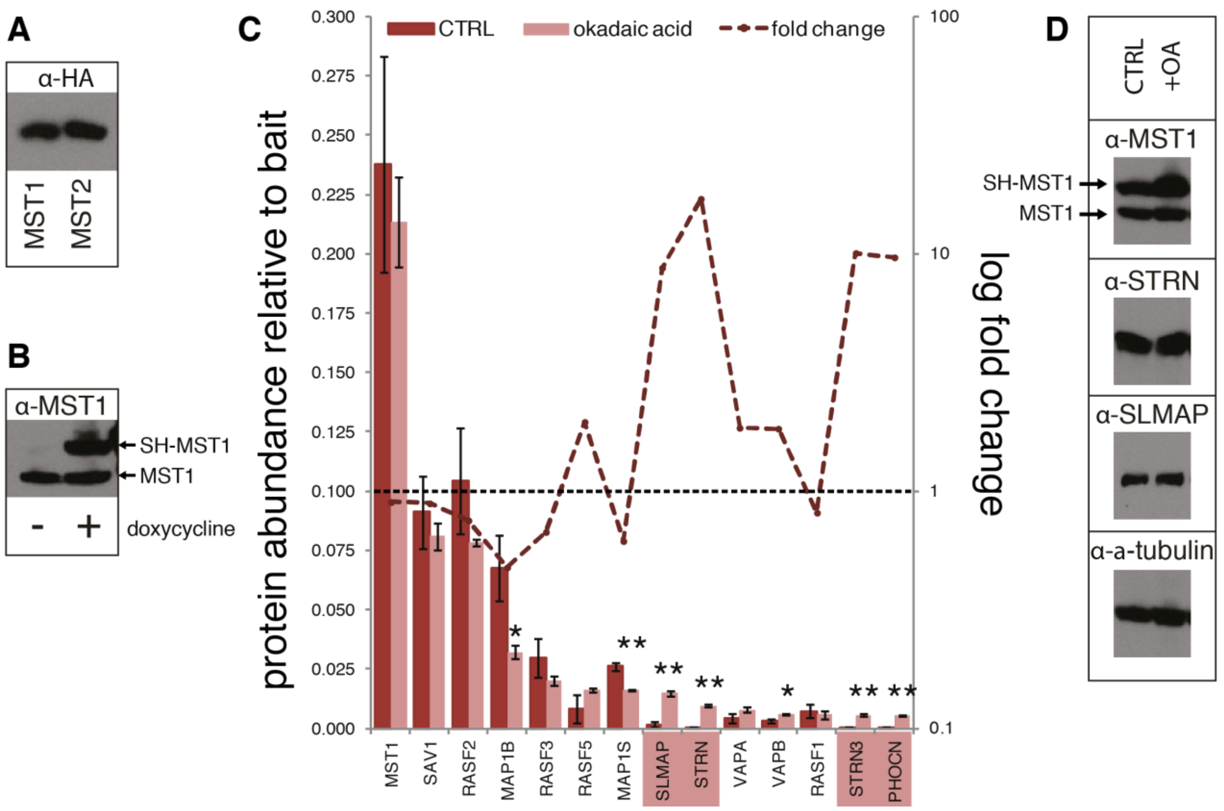

Supplementary Figure S4: MST2 binds to STRIPAK components following okadaic acid treatment.

(A) Western blot analysis of exogenous SH-MST1 and SH-MST2 with anti-HA antibody. (B) Western blot analysis of endogenous MST1 and exogenous SH-MST1 protein with anti-MST1 antibody. (C) Abundance changes of interacting proteins of MST2 upon okadaic acid stimulation. HEK293 cells expressing Strep-HA tagged MST2 were treated with 100 nM okadaic acid (OA) for 2h. Left axis represents the protein abundance relative to MST2. Right axis (log fold change; dotted line) is the logarithmic fold change of the relative abundance of proteins bound to MST2, following OA treatment. Similar to MST1 (Figure 3C), MST2 complexes contained an increased amount of STRIPAK (protein names highlighted in red) associated proteins, whereas SARAH module components only showed marginal changes. Error bars indicate standard deviation from biological triplicates. Asterisks indicate t-test statistical significance (* $p < 0.05$; ** $p < 0.01$). (D) Effects of okadaic acid treatment on expression levels of MST1, STRN and SLMAP (probed with corresponding antibody for the endogenous proteins).

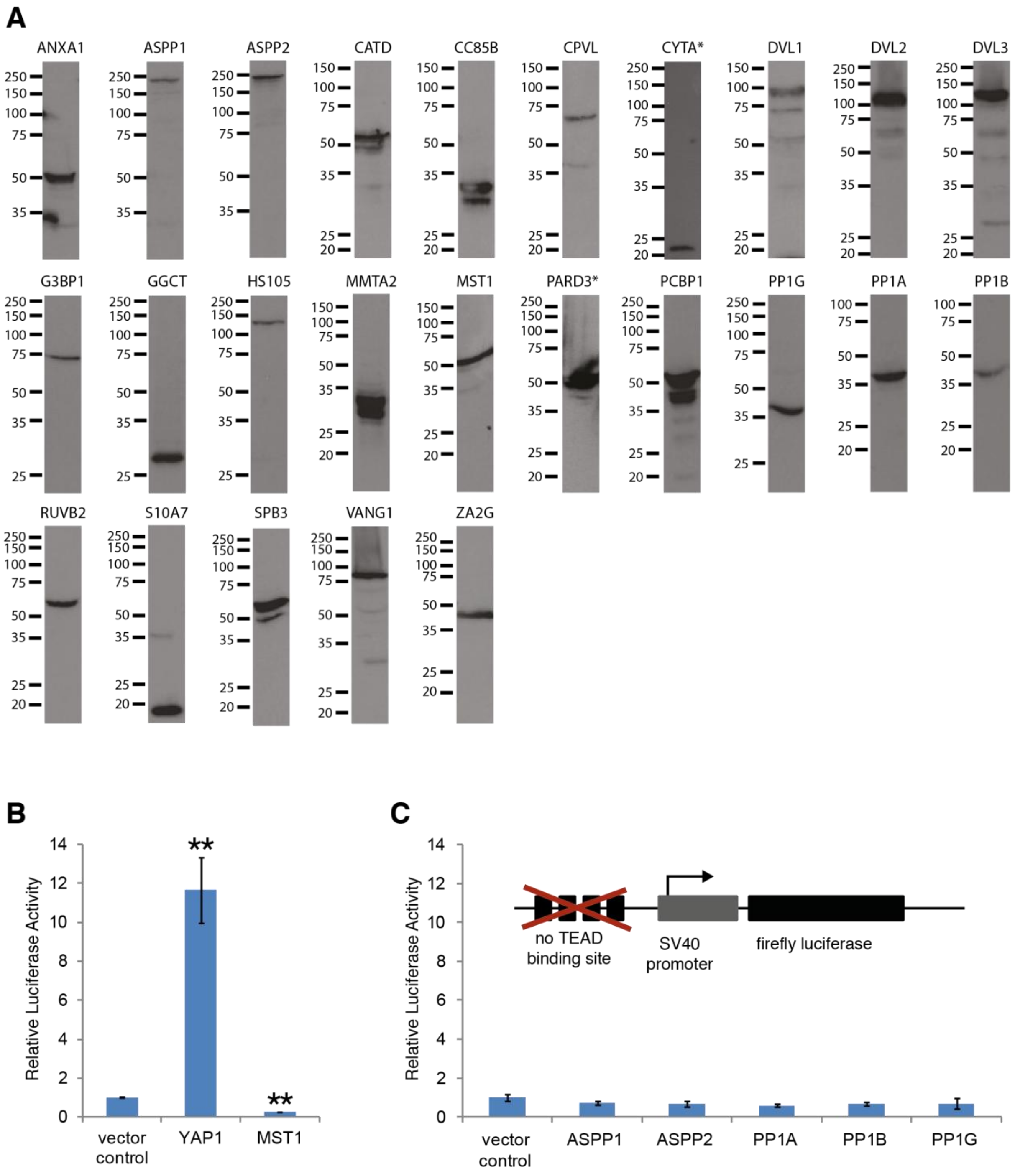

Supplementary Figure S5: Dual luciferase reporter assay of PP1-ASPP network components.

(A) Western blots of indicated Hpo network components expressed as V5-tagged proteins in transiently transfected HEK293-SH-YAP1 cells used for dual luciferase assays. Overexpressed proteins were detected with anti-V5 and anti-mouse-HRP antibodies. Asterisk (*) indicates longer exposure time. (B) Co-transfection of V5-YAP1 and V5-MST1 were used as positive and negative controls for the TEAD luciferase assay. Error bars

indicate standard deviation from biological triplicates. Asterisks indicate t-test statistical significance (* $p < 0.05$; ** $p < 0.01$). (C) ASPP2 and PP1A/G mediated transcriptional activation shown in Figure 4B require TEAD binding sites. Dual luciferase assay following transfection of indicated expression plasmids and pGL3-49 lacking TEAD binding sites.

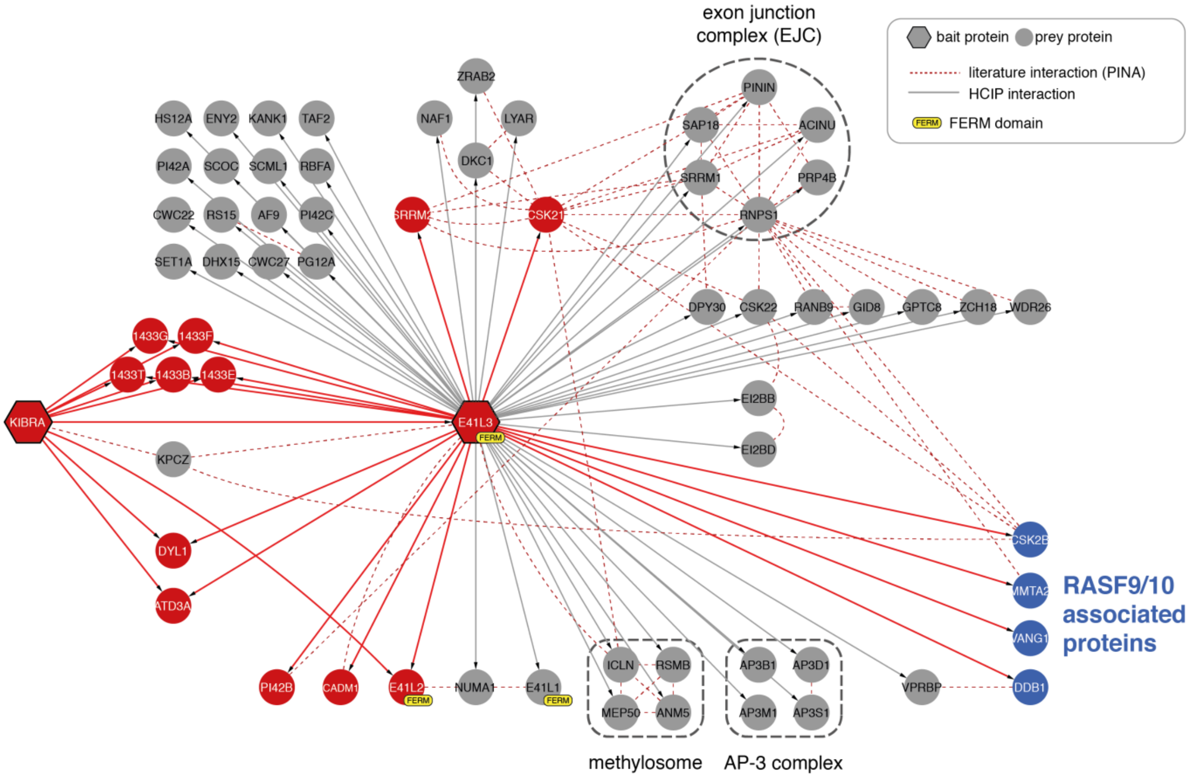

Supplementary Figure S6: The E41L3 interaction proteome.

E41L3 AP-MS revealed the most connected interactome of this study (62 HCIPs). E41L3 interacts with Hpo network component KIBRA and proteins shared with RASF9 and RASF10. Public protein interaction data for the identified prey proteins revealed associations of E41L3 with several protein complexes, such as the exon junction complex, the methylosome and the AP-3 complex. Node color corresponds to the partitioning in the modules defined in Figure 2. Proteins not allocated to specific modules are shown in grey.

Supplementary Materials and Methods

Co-immunoprecipitation and western blotting analysis

For co-immunoprecipitation and Western blotting analysis, 5 x 10^5 HEK-293 ATCC® (LGC Standards GmbH) were co-transfected with HA-tagged and V5-tagged interacting proteins. After 24h of transient expression, the cells were lysed with 500 µl of lysis buffer (50 mM HEPES pH 7.5, 150 mM NaCl, 50 mM NaF, 0.5% Igepal CA-630 (NP-40 Substitute), 200 µM Na_3VO_4, 1 mM PMSF, and 1x Protease Inhibitor mix (Sigma)) and incubated on ice for 10 min. Insoluble material was removed by centrifugation. Cleared lysates were incubated with 30 µl HA11 agarose (Sigma) for 1h at 4°C on a tube rotator. The beads were washed three times with lysis buffer and eluted with SDS sample buffer. The samples were boiled at 95°C for 5 min and separated by gel electrophoresis on 10% SDS-PAGE gels. Western blot transfer was done on nitrocellulose membranes (Whatman GmbH) using a semi-dry western blotting system (Bio-Rad). Immunoprecipitated proteins were detected with the primary monoclonal anti-HA antibodies (HA.11, Covance Inc) and anti-V5 (Life Technologies), respectively. As secondary antibody horseradish peroxidase (HRP) conjugated anti-mouse HRP (Santa Cruz Biotechnology Inc) was used for detection by enhanced chemiluminescence (ECL; Bio-Rad).

Western blotting analysis of endogenous proteins

5 x 10^5 HEK-293 ATCC® (LGC Standards GmbH) were either left untreated or induced with 1.3 µg/ml doxycline for 24 h. Where indicated, cells were treated with 100 nM okadaic acid for 2 h. The cells were lysed with 500 µl of lysis buffer (50 mM HEPES pH 7.5, 150 mM NaCl, 50 mM NaF, 0.5% Igepal CA-630 (NP-40 Substitute), 200 µM Na_3VO_4, 1 mM PMSF, and 1x Protease Inhibitor mix (Sigma)) and incubated on ice for 10 min. Insoluble material was removed by centrifugation. The proteins from the cleared lysate were denatured with SDS sample buffer and by boiling at 95°C for 5 min, and separated by gel electrophoresis. Western blotting was done using a semi-dry blotting system (Bio-Rad). Endogenous proteins levels were detected with antibodies for MST1 (Cell Signaling; Cat#: 3682S), STRN (Santa Cruz Biotechnology Inc; Cat#: sc-136084), SLMAP (Santa Cruz Biotechnology Inc; Cat#: sc-100957) and α-Tubulin (Sigma Aldrich). As secondary antibody horseradish peroxidase (HRP) conjugated anti-mouse-HRP or anti-rabbit-HRP (Santa Cruz Biotechnology Inc) was used for detection by enhanced chemiluminescence (ECL; Bio-Rad).
